# Adverse drug reactions due to opioid analgesic use in New South Wales, Australia: a spatial-temporal analysis

**DOI:** 10.1186/s40360-019-0333-7

**Published:** 2019-09-05

**Authors:** Wei Du, Shanley Chong, Andrew J. McLachlan, Lan Luo, Nicholas Glasgow, Danijela Gnjidic

**Affiliations:** 1 Research School of Population Health, Australian National University, Building 63, Eggleston Road, Acton, ACT 2601 Australia; 2 South Western Sydney Area Health Services, Sydney, Australia; 3 Sydney Pharmacy School, Faculty of Medicine and Health, The University of Sydney, Sydney, Australia; 4 Charles Perkins Centre, University of Sydney, Sydney, Australia

**Keywords:** Pharmaceutical opioid analgesics, Adverse drug reaction, Ageing, Health services

## Abstract

**Background:**

Pharmaceutical opioid analgesic use continues to rise and is associated with potentially preventable harm including hospitalisation for adverse drug reactions (ADRs). Spatial detection of opioid-related ADRs can inform future intervention strategies. We aimed to investigate the geographical disparity in hospitalised ADRs related to opioid analgesic use, and to evaluate the difference in patient characteristics between areas inside and outside the geographic clusters.

**Methods:**

We used the all-inclusive Admitted Patient Dataset for an Australian state (New South Wales, NSW) to identify patients admitted for opioid-related ADRs over a 10-year period (July 2004 to June 2014). A space-time analysis was conducted using Kulldroff’s scan statistics to identify statistically significant spatial clusters over time. Relative risk (RR) was computed with *p*-value based on Monte Carlo Simulation. Chi-square test was used to compare proportional difference in patient clustering.

**Results:**

During the study period, we identified four statistically significant geographic clusters (RRs: 1.63–2.17) during 2004–08; and seven clusters (RRs: 1.23–1.69) during the period 2009–14. While identified high-risk clusters primarily covered areas with easier access to health services, those associated with socioeconomically disadvantaged areas and individuals with mental health disorders experienced more unmet healthcare needs for opioid analgesic safety than those from the rest of the State. Older people (≥65 years and over) accounted for 62.7% of the total study population and were more susceptible to opioid-related ADRs than younger people,. In the first five-year period the clusters included a greater proportion of people with cancer in contrast to the second five-year period in which there was a lesser proportion of people with cancer.

**Conclusions:**

These results suggest that there is significant spatial-temporal variation in opioid-related ADRs and future interventions should target vulnerable populations and high-risk geographical areas to improve safer use of pharmaceutical opioid analgesics.

## Background

Australia has a high and rapidly increasing use of pharmaceutical opioids [1–3]. Approximately 3 million Australians have at least one prescription of opioid analgesic dispensed annually, most commonly codeine in combination with paracetamol, or oxycodone [1]. Australia’s total annual opioid analgesic consumption is ranked 4th (per capita) globally, with a 4-fold increase over the last decade [2]. The cost to the Australian Federal Government of subsidised opioid analgesics has increased more than 30-fold over the last two decades ($AUD 271 M in 2012) [3]. In addition to the Government subsidy, non-cancer pain is another major driver of increased pharmaceutical opioid analgesic use [4–6]. Adverse outcomes associated with pharmaceutical opioid analgesic use, including premature deaths, are on the rise [7–9].

Opioid prescribing practices demonstrate substantial geographical variation [10–12]. There has been an increase in research applying spatial statistics focusing on opioid analgesic use disorders and overdoses [11, 13]. However much less is known about geographical patterns of adverse drug reactions (ADR), another common concern in relation to therapeutic opioid analgesic use [14–18]. Spatial scan statistics are widely applied in population health. These techniques identify potential geographic clusters and compare the population based relative risk for an event of interest [19]. Considering nearly half (45%) of hospitalised ADRs are preventable [20], the application of spatial scan statistics has the clear advantage in ADR surveillance to inform development of intervention strategies.

Using an all-inclusive hospital inpatient dataset, this study aimed 1) to investigate whether there were any significant geographical clusters of opioid analgesic-related ADRs; and 2) to compare patient characteristics inside clusters with patients outside each identified cluster. The ultimate goal is to inform potential prevention strategies that may reduce ADR due to pharmaceutical opioid analgesic use.

## Methods

### Data sources

We used the New South Wales (NSW) Admitted Patient Data Collection (APDC) over the 10-year period from July 2004 to June 2014, which is a complete census of hospital separations in NSW, Australia. Maintained by the NSW Health Department, the APDC comprises information and activities of admitted patients including demographic and clinical information from all public and private hospitals in NSW. Medical reasons for hospital admission were coded at the time of discharge using the 10th version of International Statistical Classification of Diseases and Related Health Problems Australian Modification (ICD-10 AM) [21].

Based on the data use agreement with NSW Health Department, we extracted demographic and clinical information for each de-identified separation record including the patient’s age, sex, residential postcode, private insurance status, and up to 53 medical diagnoses. The Australian National University Science & Medical Delegated Ethics Review Committee approved this study (#2016/030), with the need for consent waived given the use of de-identifiable data for secondary analysis.

### Definitions

We used ICD-10 AM codes (Y45.0 Opioids and related analgesics) from *Chapter ‘External causes of morbidity and mortality’* to select the hospitalised ADR incidents caused by opioids ‘in proper therapeutic use’. [21] Data on specific opioid analgesics were not available and hereinafter ADRs refer to any use of opioid analgesics related adverse drug reactions in general. Similarly, we considered comorbid conditions in terms of hospitalised major injury and disease groups widely reported as leading causes of death or clinical significant pain, i.e., coronary heart diseases (ICD-10 AM codes I20–I25), cerebrovascular diseases (I60–I69), cancers (C00–C97), brain degenerative disorders in particular dementia and Alzheimer disease (F01,F03, G30), chronic obstructive respiratory diseases (J40–J44), and diabetes mellitus (E10-E14), and osteoarthritis (M15-M19). There were multiple updates to the ICD-10 AM during the study periods, which did not affect these codes.

Because the APDC consisted of de-identified episodes of hospital care, we only considered cases admitted for acute care based on their admission status being urgent to reduce the impact of multiple counting of the same ADR event. We excluded inpatients with unknown age and sex. We categorised age into five groups, i.e., < 18, 18–44, 45–64, 65–84, or 85+ years; sex as male or female; private insurance as yes or no; marital status as single or others; socioeconomic status as 1st (most disadvantaged), 2nd, 3rd, 4th, or 5th (least disadvantaged) quintile using the postcode based ‘Index of Relative Socioeconomic Disadvantage’, [22] which summarises a range of economic and social conditions specific to an area with a lower score indicating a relatively greater disadvantage; rurality of residence as urban or rural using the geographic Accessibility/Remoteness Index of Australia plus (ARIA+) index quantifying remoteness in terms of travelling distance to different size of population-adjusted service centres [23]; convenience to pharmacies as more convenient (i.e., most accessible to a pharmacy) or rather less based on the composite Pharmacy ARIA (PhARIA) index measuring geographic remoteness (represented by ARIA+) as well as professional isolation (travelling distance to the five closest pharmacies) [24]; severity of comorbidities using revised Charlson Comorbidity Index [25], as minor (score equal to 0), moderate (score equal to 1 or 2) or severe (score ≥ 3); and disposition status as either died at hospital, or alive at discharge.

### Statistical analysis

We carried out the space-time analysis using SaTScan v9.6 [26]. Kulldroff’s scan statistics were used to identify the presence of statistically significant spatial clustering of the hospitalised opioid analgesic-related adverse reactions across a total of 570 NSW Australian post-code areas. This method progressively moves a cylindrical scan window in space and time and calculates the observed and expected number of cases for each post-code area in this study [27]. For each post-code area, the radius of the scan window varied continuously in size from zero to the 20% of the study population to account for the small number of opioid-related adverse reactions at postcode level. This window size was selected to generate high risk areas that make sense from a health system perspective (e.g. local government areas), as these could then become the focus for locally delivered responses. In addition we re-ran the model using window sizes from 10 to 25% for every 5% increase and found the results to be very similar. We also examined the clusters year by year and found the locations and the sizes of the clusters varied greatly before and after 2009. Therefore we divided the data into two five-years periods, 2004–05 to 2008–09 and 2009–10 to 2013–14. For count event data, Poisson model was applied with population at postcode level adjusted and relative risks (RRs) calculated for specific locations of clusters. We used the likelihood ratio test to evaluate the statistical significance of an identified cluster, with the *p*-value generated using Monte Carlo Simulation [28]. The number of permutations was set to 999 to ensure adequate power for defining clusters, and a *p*-value < 0.05 was set as statistically significant. The scan window with the maximum likelihood value was the most likely cluster. For secondary likely clusters, the non-overlapping option was selected. Mantel-Haenszel Chi-Square test was used to compare the proportional differences in the characteristics of the study population from identified clusters to those from the remaining regions of NSW.

## Results

A total of 26,776 opioid-related ADR incident cases (reflecting the real incidence in the NSW residential population) in 570 post-code areas from NSW were hospitalised for acute care from 2004 to 05 to 2013–14, demonstrating an overall increasing trend over time. Of these 59.3% (*n* = 15,887) were females, 62.7% (*n* = 16,802) were aged 65 years and over, and almost a quarter (*n* = 5966) lived in the most socioeconomically disadvantaged areas (Table 1). Approximately 22.5% of the study population were admitted for injurious conditions (e.g. fractures of femur or rib) as the primary reason for admission, followed by conditions of the digestive systems (e.g., constipation or intestinal obstruction) (17.2%) and less well-defined bodily symptoms and signs (15.4%).

**Table 1:** Characteristics of study population (*n* = 26,776)

	Number (%)
Opioid-related ADRs by Year	
2004–05	1215 (4.5)
2005–06	1464 (5.5)
2006–07	1756 (6.6)
2007–08	1774 (6.6)
2008–09	2194 (8.2)
V2009–10	2697 (10.1)
2010–11	3242 (12.1)
2011–12	3681 (13.7)
2012–13	4068 (15.2)
2013–14	4685 (17.5)
Age (years) of people involved in Opioid-related ADRs	
< 18	614 (2.3)
18–44	4012 (15.0)
45–64	5348 (20.0)
65–84	11,388 (42.5)
85+	5414 (20.2)
Gender	
Male	10,889 (40.7)
Female	15,887 (59.3)
Marital status	
Single	13,826 (51.6)
Others	12,950 (48.4)
Private Insurance	
Yes	7754 (29.0)
No	19,022 (71.0)
Socioeconomic disadvantage	
Most (1st quintile)	5966 (22.3)
Others (2nd to 5th quintile)	20,810 (77.7)
Rurality of residence	
Urban	25,282 (94.4)
Rural	1494 (5.6)
Convenient access to pharmacy	
More	24,087 (90.0)
Less	2689 (10.0)
Severity of comorbidities	
Minor	15,835 (59.1)
Moderate	5845 (21.8)
Severe	5096 (19.0)
Disposition status	
Alive	25,735 (96.1)
Dead	1041 (3.9)

### Spatial temporal pattern

**Figure 1:**
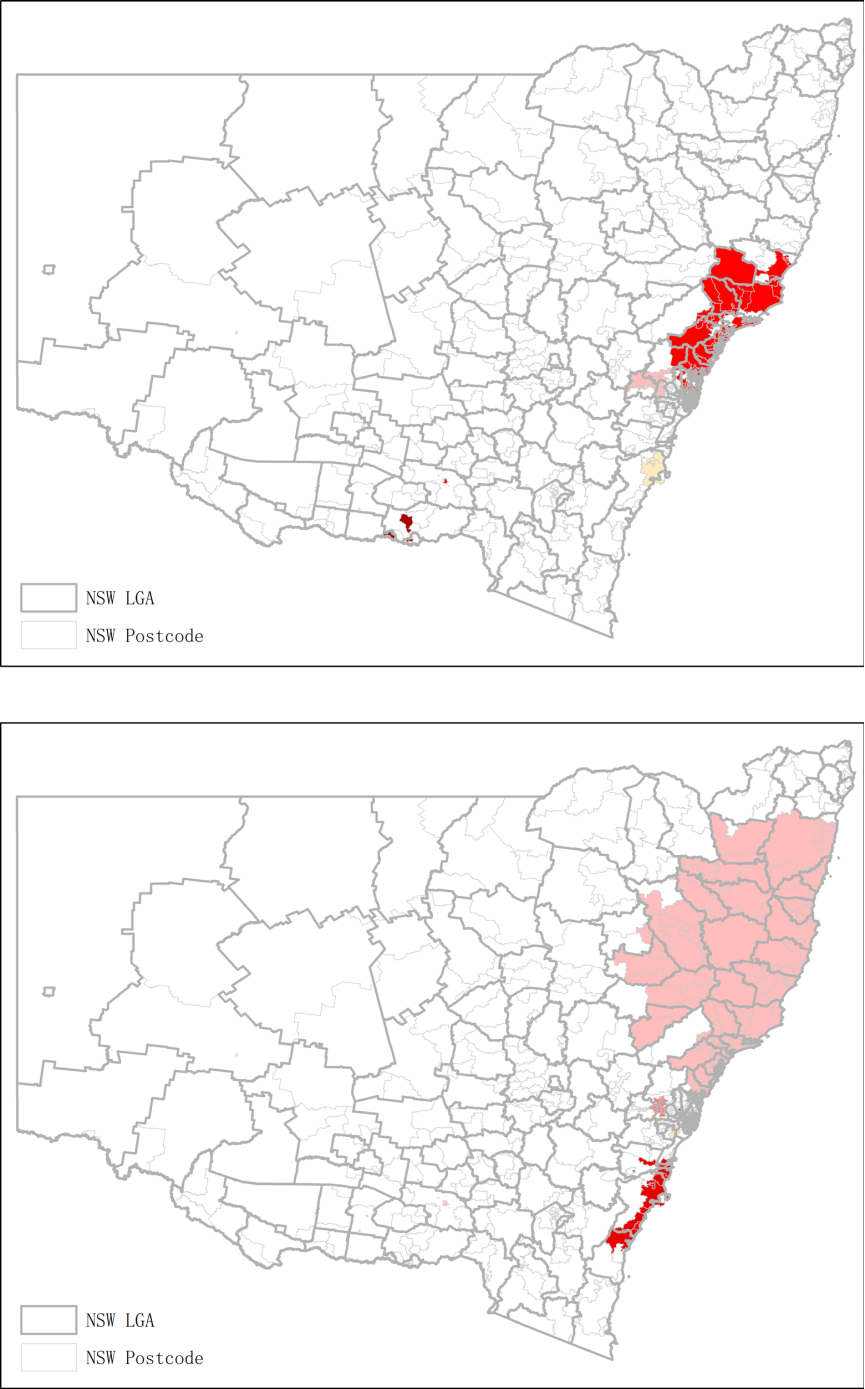
1.1 Clusters of opioid-related ADRs for hospitalisation in NSW (Period: 2004-08). 1.2 Clusters of opioid-related ADRs for hospitalisation in NSW (Period: 2009-14)

**Figure 2:**
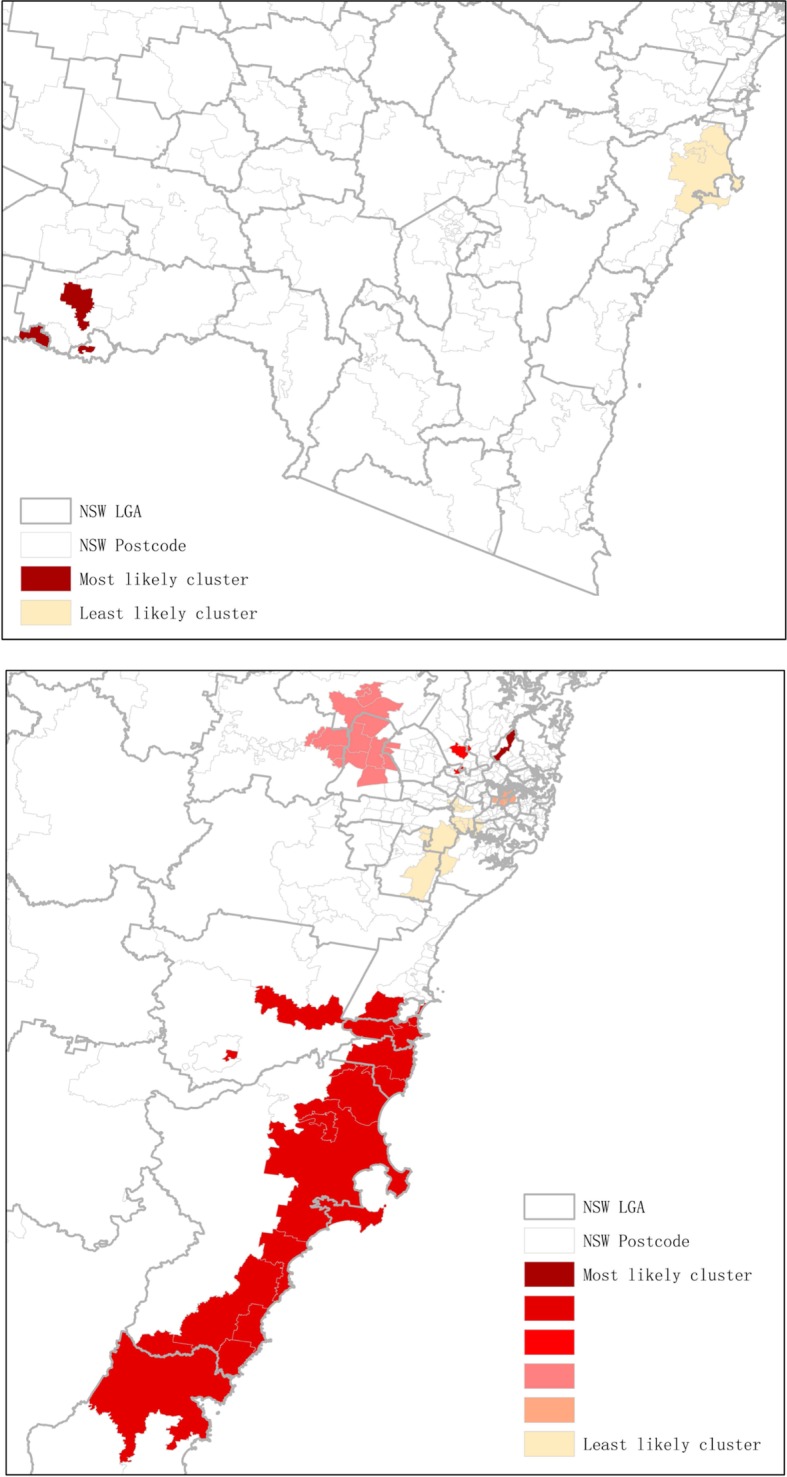
2.1 Most and least likely clusters of opioid-related ADRs for hospitalisation in NSW (Period: 2004-08). 2.2 Most and least likely clusters of opioid-related ADRs for hospitalisation in NSW (Period: 2009-14)

During the five-year period of 2004–05 to 2008–09, four statistically significant clusters of opioid-related ADRs were identified with RRs in the range of 1.63 to 2.17 (Table 2). These clusters comprised 38.7% of total incidents (*n* = 8403). While the second and third likely clusters were closely connected, the other two clusters were primarily isolated in regional NSW (Figs. 1 and 2).

**Table 2:** Clusters of opioid-related adverse drug reactions for hospitalisation in NSW

Cluster	No. post-code areas	Observed cases	Expected cases	Log likelihood ratio	Relative risk	*p*-value
2004–05 to 2008–09						
Most likely cluster	3	49	22.69	11.45	2.17	0.010
2nd	109	2599	1605.02	335.21	1.90	0.001
3rd	22	465	278.70	53.89	1.71	0.001
Least likely cluster	3	142	87.66	14.33	1.63	0.001
2009–10 to 2013–14						
Most likely cluster	1	94	55.61	10.99	1.69	0.020
2nd	14	1015	635.27	99.99	1.63	0.001
3rd	2	201	127.29	18.26	1.59	0.001
4th	13	696	487.26	40.64	1.45	0.001
5th	121	4526	3452.59	191.41	1.41	0.001
6th	10	434	312.59	21.42	1.40	0.001
Least likely cluster	13	527	430.18	10.42	1.23	0.033

During the five-year period of 2009–10 to 2013–14, seven statistically significant clusters were identified with RRs in the range of 1.23 to 1.69 (Table 2). These clusters comprised 40.8% of total incidents (*n* = 18,373), and demonstrated an increasing incidence in comparison to the previous five-year period. More clusters were identified in the metropolitan areas and the clusters identified in the first five-year period along the coastlines were spreading and covering more local government areas (Figs. 1 and 2).

### Cluster characteristics

In-hospital mortality was greater within the cluster patients compared to non-cluster patients in the first five-year period, but this difference was not evident in the second five-year period (Table 3). Similarly the identified clusters had a higher proportion of patients from urban areas or places with more convenient access to pharmacies during the first five-year period, and this proportional difference was narrowing over time with a similar higher proportion of incidents (> 90%) having convenient access to pharmacy between those clustered in the identified regions and the rest of NSW.

**Table 3:** Characteristics of study population from identified clusters in comparison to those from the remainder regions of NSW

	2004–05 to 2008–09			2009–10 to 2013–14		
	Clusters n (%)	Non-clusters n (%)	X^2^ *p*-value	Clusters n (%)	Non-clusters n (%)	X^2^ *p*-value
Total	3255 (100)	5148 (100)		7493 (100)	10,880 (100)	
Age group (years)						
< 18	77 (2.4)	141 (2.7)	0.005	161 (2.1)	235 (2.2)	0.396
18–44	509 (15.6)	820 (15.9)		1098 (14.7)	1585 (14.6)	
45–64	625 (19.2)	1073 (20.8)		1403 (18.7)	2247 (20.7)	
65–84	1404 (43.1)	2263 (44.0)		3252 (43.4)	4469 (41.1)	
85+	640 (19.7)	851 (16.5)		1579 (21.1)	2344 (21.5)	
Gender						
Male	1401 (43.0)	2032 (39.5)	0.001	3006 (40.1)	4450 (40.9)	0.288
Female	1854 (57.0)	3116 (60.5)		4487 (59.9)	6430 (59.1)	
Marital status						
Single	1605 (49.3)	2616 (50.8)	0.178	3946 (52.7)	5659 (52.0)	0.386
Others	1650 (50.7)	2532 (49.2)		3547 (47.3)	5221 (48.0)	
Private insurance						
Yes	1043 (32.0)	1269 (24.7)	< 0.001	1941 (25.9)	3501 (32.2)	< 0.001
No	2212 (68.0)	3879 (75.3)		5552 (74.1)	7379 (67.8)	
Socioeconomic disadvantage						
1st (most)	589 (18.1)	1255 (24.4)	< 0.001	2008 (26.8)	2114 (19.4)	< 0.001
2nd	639 (19.6)	1114 (21.6)		1897 (25.3)	2050 (18.8)	
3rd	609 (18.7)	997 (19.4)		1675 (22.4)	2092 (19.2)	
4th	660 (20.3)	963 (18.7)		1385 (18.5)	1955 (18.0)	
5th (least)	758 (23.3)	819 (15.9)		528 (7.0)	2669 (24.5)	
Rurality of residence						
Urban	3245 (99.7)	4649 (90.3)	<.0001	7251 (96.8)	10,137 (93.2)	< 0.001
Rural	10 (0.3)	499 (9.7)		242 (3.2)	743 (6.8)	
Convenient access to pharmacy						
More	3115 (95.7)	4399 (85.5)	< 0.001	6752 (90.1)	9821 (90.3)	0.727
Less	140 (4.3)	749 (14.5)		741 (9.9)	1059 (9.7)	
Disposition status						
Death	196 (6.0)	196 (3.8)	< 0.001	276 (3.7)	373 (3.4)	0.357
Alive	3059 (94.0)	4952 (96.2)		7217 (96.3)	10,507 (96.6)	
Severity of comorbidities						
Minor	1760 (54.1)	3023 (58.7)	0.005	4467 (59.6)	6585 (60.5)	0.995
Moderate	744 (22.9)	1073 (20.8)		1723 (23.0)	2305 (21.2)	
Severe	751 (23.1)	1052 (20.4)		1303 (17.4)	1990 (18.3)	
Clinical conditions^a^						
CHD	282 (8.7)	381 (7.4)	0.037	281 (3.8)	396 (3.6)	0.696
Cancer	578 (17.8)	795 (15.4)	0.005	983 (13.1)	1566 (14.4)	0.014
BDD	199 (6.1)	211 (4.1)	< 0.001	373 (5.0)	379 (3.5)	< 0.001
COPD	234 (7.2)	326 (6.3)	0.125	421 (5.6)	511 (4.7)	0.005
CVD	96 (2.9)	128 (2.5)	0.199	140 (1.9)	208 (1.9)	0.832
Diabetes	454 (13.9)	685 (13.3)	0.402	950 (12.7)	1402 (12.9)	0.679
Osteoarthritis	147 (4.5)	201 (3.9)	0.170	187 (2.5)	279 (2.6)	0.771
Number of mental disorders						
None	2436 (74.8)	4115 (79.9)	< 0.001	5467 (73.0)	8346 (76.7)	< 0.001
Single	674 (20.7)	869 (16.9)		1699 (22.7)	2232 (20.5)	
Multiple (≥2)	145 (4.5)	164 (3.2)		327 (4.4)	302 (2.8)	

^a^Selected clinical conditions including coronary heart diseases (CHD), cancer, brain degenerative disorders (BDD), chronic obstructive respiratory diseases (COPD), cerebrovascular diseases (CVD), diabetes, and osteoarthritis, were compared between those with and without a condition, respectively

During the first five-year period, the identified clusters had a significantly higher proportion of patients with severe comorbidities, or holding private insurance cover, or living in less socioeconomic disadvantaged areas, whereas during the second five-year period, there was a turnaround in these proportions with identified clusters comprising fewer patients with severe comorbidities, and more patients from more socioeconomic disadvantaged areas or without private insurance cover.

Cancers and diabetes accounted for the majority of the selected clinical conditions in the study population. Brain degenerative disorders and mental disorders were over-represented in those from the identified clusters in comparison to those from the rest of NSW over the 10-year study period. While proportionally more patients with cancer or coronary heart disease were observed in those from the identified clusters during the first five-year period, there appeared proportionally fewer people with cancer or coronary heart disease during the second five-year period..

## Discussion

This study found substantial variability in space and time with respect to the occurrence of opioid-related ADRs for hospitalisation in NSW during the period of 2004 to 2014. We observed an increasing number of cases year on year in the study population, which was consistent with previous Australian and international findings that point to an increasing healthcare burden arising from opioid analgesic use [29–31]. This highlights the importance of developing appropriate intervention strategies to address this. The study demonstrated spatio-temporal variation with earlier opioid-related ADRs being clustered within post-code areas located in both metropolitan and regional local health districts, and more recent clusters spreading and covering more metropolitan and inner regional areas of NSW, coincident with high rates of prescriptions being dispensed for opioid analgesics in those areas [32]. Our study demonstrated the utility of this approach. Future studies could build on this approach and include other data such as number of visits to general practitioners, specialists, psychiatrists, pharmacists, and allied health professionals to further elaborate opioid-related ADR spatio-temporal variation to assist the development of appropriate policy and health service interventions.

We observed there were more patients from urban areas with opioid-related ADRs in both five-year time periods. More convenient access to pharmacies was associated with an increase in opioid-related ADRs in the earlier five-year period, but not the later one. We found a potential shift over time in opioid-related ADRs towards more socioeconomically disadvantaged population groups. Previous studies point to a potential link between greater health service utilisation and opioid-related adverse events [33–35]. Our findings raise questions regarding potential unmet healthcare needs arising through a general lack of access to evidence-based pain management services and this being compounded in socioeconomically disadvantaged population groups. Increasing both health professional and targeted community focused educational activities in relation to appropriate prescription and use of pharmaceutical opioid analgesics should help to reduce these potentially preventable events.

The increase of opioid-related ADRs among older patients is of major concern, and is consistent with previous findings that show opioid-related adverse outcomes increase with age [36, 37]. Older adults are more likely to experience adverse outcomes from pharmaceutical opioid use due to changes in their metabolic processes, highly prevalent co-morbidities, and the concurrent use of multiple medications [38–42]. Lower rates of non-medical opioid analgesic use among older adults compared with younger people have also been observed [43]. With regard to the significant increase in the use of prescription opioid analgesics in older people [44], second-line prescription opioids have been commonly used to initiate pain management in this patient population [45], as well as in those living with mental health problems [46]. For both these patient populations there is an increased risk of ADRs [47–50]. Premature deaths in relation to inappropriate use of pharmaceutical opioid analgesics in the United States have been increasing since 2006 in those who aged 60 years and above and have exceeded those aged under 60 year since 2012 [43]. Emerging evidence also indicates pharmaceutical opioid use in older people is associated with elevated risk of cardiovascular disease mortality compared to non-users [16], and with increased total mortality and hospitalisation among arthritis patients [17]. Facing an ageing population and the vulnerability of older people to opioid-related ADRs, risk mitigation strategies should be implemented to ensure that the potential benefits of any opioid prescription outweigh the risks. Our study reinforces the importance of this both to reduce harm to elderly patients arising from ADRs and reduce associated health care costs.

While prescribing opioids for the treatment of cancer pain is appropriate, it is still a significant contributor to the occurrence of adverse outcomes [51], with up to one fifth of cancer patients experiencing intolerable adverse events [52]. There is limited evidence of benefit for many non-cancer pain conditions such as low back pain [53], and prescribing opioids for non-cancer pain remains controversial [54, 55]. For example, use in the treatment of pain caused by diabetic neuropathy is deemed inappropriate and should be avoided [56]. Noting these studies, we observed an increasing pattern of opioid-related ADRs in people with cancer or living with diabetes, which between them accounted for the majority of cases with severe conditions during the study period. We found the occurrence of opioid-related ADRs was common in those with minor comorbidities with 54% of patients in the earlier five-year period and nearly 60% of patients in the second five-year period experiencing ADRs. These observed patterns may relate to an overall increasing use of opioid analgesics in healthcare settings [1–3], and/or lack of accredited multidisciplinary services for chronic pain in those local health districts in regional NSW during the study period [57], with greater use of potentially inappropriate opioid analgesics in various clinical scenarios [49, 50].

We observed the high-risk clusters comprised more people living with mental health problems than the rest of NSW. This may indicate a lack of awareness of the risk of pharmaceutical opioid analgesic use in mental health care settings. While use of prescription sedative-hypnotics predicts persistence in pharmaceutical opioid use [58], their concurrent use would potentially create a lethal combination.

With both more kinds of pharmaceutical opioid analgesics being made available and more potent agents within these, inappropriate use of these drugs and consequent greater burden of opioid-related ADRs in the healthcare systems is likely to occur. Given opioid analgesics should be used with great care for cancer and non-cancer pain [14, 53], and the evidence base informing therapeutic risk ratios is dynamic, regular updates of guidelines is vital to underpin opioid prescribing, dispensing and administration in all clinical settings. In addition, healthcare provider organisations should include clinical audits in regard to opioid-related ADRs and develop, implement, and evaluate targeted intervention strategies to improve safer use of these agents. Other strategies may include but not be limited to the establishment of multidisciplinary and comprehensive chronic pain services, and ensuring opioid-related ADR risks are understood in all parts of the health system including patient groups, general practice, community pharmacy, residential aged care, and acute care settings. Continuing professional development for health care practitioners is key, as is raising community awareness of the role these agents may safely play.

Global pharmacovigilance efforts are underway to address the opioid epidemic. Our study describes geographical dimensions to the challenge in NSW and may allow some priorities to be set taking into account the observed geographical variation. Our study has some limitations. First, the use of administrative inpatient data with restricted contextual information limits our ability to identify patient-level causal factors in relation to the observed spatial-temporal variation. In this study we were unable to measure opioid analgesic prescription, dispensing, and administration patterns and relate these to ADRs in specific clusters. Second, heterogeneity of health service provision in relation to use of pharmaceutical opioid analgesics between geographic regions may contribute in part to observed spatial-temporal patterns in NSW. For example, hospital admission practices may vary across different regions. Our findings indicate a need for future investigation of local healthcare policy and community programs that may influence appropriate use of pharmaceutical opioids. Third, measurement errors may occur while using the ICD-coded data with some ADR cases being underreported in the administrative data. Considering the APDC data has undergone routine data quality checks, we deemed the impact due to such errors minimal and unlikely to explain the observed spatial-temporal patterns. This study focuses on the adverse events associated with pharmaceutical opioid use leading to acute admission to hospital. It is important to acknowledge that good clinical practice will continue to make appropriate use of these agents based on evidence and careful consideration of the risk to benefit ratio for a given patient.

## Conclusion

These results suggest that there is significant spatial-temporal variation in opioid-related ADRs in NSW. Older people, people with mental health conditions, people with less severe comorbidities, and people from more socioeconomically disadvantaged areas were susceptible to opioid-related ADRs serious enough for acute hospital care. Strategies should be developed, implemented and evaluate to address opioid-related ADRs, where possible taking account of the geographic and temporal variations demonstrated.

## Data Availability

No additional data are available.

## Abbreviations

ADRs: Adverse drug reactions
ARIA+: Accessibility/Remoteness Index of Australia plus
ICD-10 AM: 10th version of International Classification of Diseases Australian Modification
NSW: New South Wales
PhARIA: Pharmacy ARIA
RR: Relative risk
